# Treating ICB-resistant glioma with anti-CD40 and mitotic spindle checkpoint controller BAL101553 (lisavanbulin)

**DOI:** 10.1172/jci.insight.142980

**Published:** 2021-09-22

**Authors:** Vassilis Genoud, Felipe I. Espinoza, Eliana Marinari, Viviane Rochemont, Pierre-Yves Dietrich, Paul McSheehy, Felix Bachmann, Heidi A. Lane, Paul R. Walker

**Affiliations:** 1 Translational Research Center for Hemato-Oncology, Faculty of Medicine, University of Geneva, Geneva, Switzerland.; 2 Department of Oncology, University Hospitals of Geneva, Geneva, Switzerland.; 3 Basilea Pharmaceutica International Ltd., Basel, Switzerland.

**Keywords:** Immunology, Oncology, Brain cancer, Cancer immunotherapy, Innate immunity

## Abstract

Glioblastoma is a highly malignant brain tumor with no curative treatment options, and immune checkpoint blockade has not yet shown major impact. We hypothesized that drugs targeting mitosis might affect the tumor microenvironment and sensitize cancer cells to immunotherapy. We used 2 glioblastoma mouse models with different immunogenicity profiles, GL261 and SB28, to test the efficacy of antineoplastic and immunotherapy combinations. The spindle assembly checkpoint activator BAL101553 (lisavanbulin), agonistic anti-CD40 antibody, and double immune checkpoint blockade (anti–programmed cell death 1 and anti–cytotoxic T lymphocyte–associated protein 4; anti–PD-1 and anti–CTLA-4) were evaluated individually or in combination for treating orthotopic GL261 and SB28 tumors. Genomic and immunological analyses were used to predict and interpret therapy responsiveness. BAL101553 monotherapy increased survival in immune checkpoint blockade–resistant SB28 glioblastoma tumors and synergized with anti-CD40 antibody, in a T cell–independent manner. In contrast, the more immunogenic and highly mutated GL261 model responded best to anti–PD-1 and anti–CTLA-4 therapy and more modestly to BAL101553 and anti-CD40 combination. Our results show that BAL101553 is a promising therapeutic agent for glioblastoma and could synergize with innate immune stimulation. Overall, these data strongly support immune profiling of glioblastoma patients and preclinical testing of combination therapies with appropriate models for particular patient groups.

## Graphical abstract

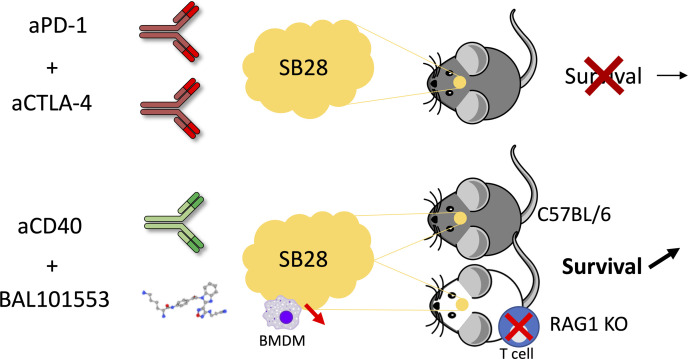

## Introduction

Glioblastoma (GBM), the most prevalent primary brain tumor, is a particularly heterogeneous and aggressive tumor, with a poor prognosis of less than 2 years of expected survival at diagnosis (1). Current standard of care comprises surgery followed by radiotherapy and temozolomide (TMZ), with no improved treatment protocols for over a decade (2). For other cancer indications, immunotherapies have brought unprecedented clinical benefits for patients (3), and there is hope that similar treatment approaches could be adapted for GBM.

Although antitumor immunity can employ diverse effector mechanisms, clinical cancer immunotherapy has become dominated by different immune checkpoint blockade (ICB) strategies, with programmed cell death 1/programmed cell death ligand 1 (PD-1/PD-L1) and cytotoxic T lymphocyte–associated protein 4 (CTLA-4) blocking antibodies (Ab) already approved for multiple indications (4). Nevertheless, it is not clear whether it is a realistic objective to extend ICB to a majority of cancer indications, as most patients are still unresponsive, with lack of T cell infiltration (5) or low mutational load (6) being linked to this ICB resistance. For GBM, most tumors exhibit a low mutational load with few infiltrating T cells, and they can be described as immunologically “cold” tumors (7). Mutational load is linked to immunotherapy through neoepitopes that originate from mutated proteins and that are highly immunogenic (8) but need to be presented to T cells by MHC class I (MHC-I), which is downregulated in human GBM (9). Moreover, GBM generates a highly immunosuppressive tumor microenvironment (TME), with high infiltration of potentially protumor regulatory T cells (Treg) and M0/M2 macrophages (10, 11).

In several preclinical studies, “cold” tumors have been sensitized to ICB monotherapy, by combining different treatment strategies such as antineoplastic agents, or by targeting multiple immune checkpoints (12), with some treatment combinations also tested in GBM models (13, 14). However, as we have previously shown (15), the baseline ICB response of mouse GBM models can vary considerably, likely mirroring very different patient groups. Therefore, when investigating combination treatment for GBM in studies that will translate to clinical application, the choice of mouse model is crucial. The GL261 glioma mouse model is highly sensitive to ICB (15, 16) and may recapitulate responses of certain, but rare, GBM patients, whereas the SB28 model will better represent the immunotherapy sensitivity of the majority of human GBM (15).

In this study we used both SB28 and GL261 mouse GBM models to explore therapeutic combinations, including ICB, with a focus on the more stringent and challenging SB28 model. In view of the major myeloid cell infiltration but minor T cell infiltration of SB28 tumors (15), we anticipated that both immune compartments would require modulation to achieve therapeutic effect. We hypothesized that drugs inducing mitotic spindle checkpoints would induce genomic instability in tumor cells, as a first step toward sensitizing them to ICB. Hence, we investigated the use of BAL27862 (avanbulin) and its prodrug BAL101553 (lisavanbulin) (17), a novel microtubule-disrupting agent (18), whose activity is dependent on activation of the mitotic spindle assembly checkpoint (SAC) (19), which has high blood-brain barrier penetrance (20) and activity in orthotopic GBM models (21). BAL101553 is currently being evaluated in phase I/IIa trials for GBM as monotherapy or in combination with radiotherapy (ClinicalTrials.gov identifiers NCT03250299 and NCT02490800). This compound showed in vitro and in vivo activity in the 2 models used, SB28 and GL261. Immunotherapeutic strategies alone, including anti–PD-1 (aPD-1) and anti–CTLA-4 (aCTLA-4) ICB, or agonistic anti-CD40 (aCD40) Ab, acting on macrophages and DCs, were not efficacious in the stringent SB28 model, but a synergistic effect leading to prolonged survival was obtained with a combination of aCD40 and BAL101553. Analysis of SB28 mutational load and the immune infiltrate suggested that this combined therapy might be able to function independently of T cells, which was confirmed by reproducing the therapeutic effect in T cell– and B cell–deficient mice. The same treatment strategies were also tested in the more immunogenic GL261 model, which revealed a favorable outcome when BAL101553 was combined with aCD40, but not with ICB. Overall, these results indicate that, with an optimal combination of treatment modalities, a synergistic effect of selected antineoplastic drugs and immunotherapy agents can be obtained even in a stringent preclinical model of GBM.

## Results

### BAL27862 inhibits SB28 glioma cell proliferation in vitro and stimulates release of high mobility group box 1.

**Figure 1:**
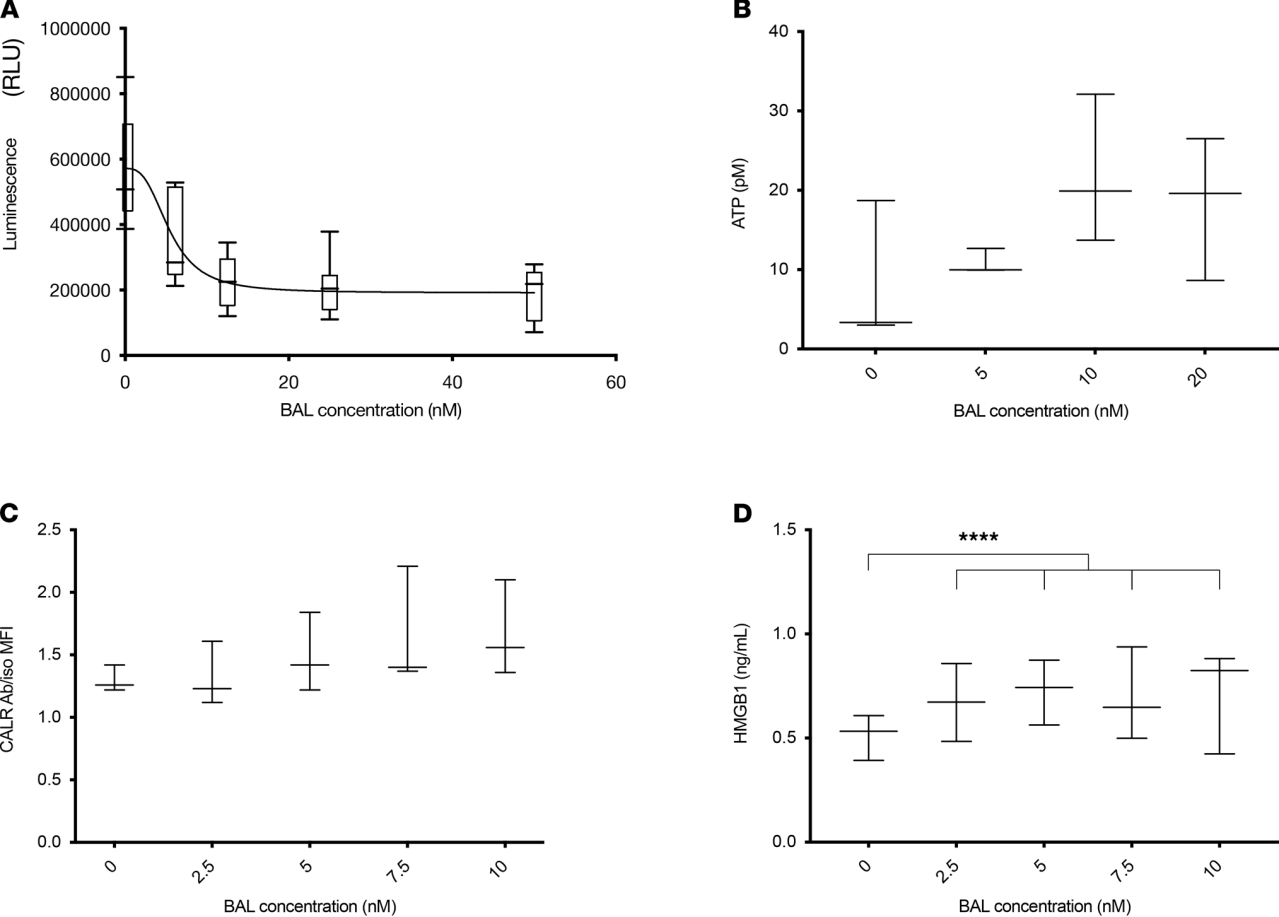
BAL27862 affects the number of viable SB28 cells in vitro but is a modest immunogenic cell death inducer. (**A**) Number of viable SB28 cells based on total ATP content by luminescent (in relative light units) cell viability/proliferation assay; SB28 sensitivity to BAL27862 (BAL) corresponds to an IC_50_ of 5.466 nM. (**B**–**D**) Evaluation of BAL27862 induction of key ICD features: (**B**) extracellular ATP release, (**C**) membrane translocation of calreticulin (CALR, calculated as MFI ratio), (**D**) HMGB1 release. Statistics: 2-way ANOVA with Tukey’s multiple-comparison test. ****: *P* < 0.0001. Box-and-whisker plot with the bounds of the box representing lower and upper quartiles, the line within the box showing the median, and the whiskers showing minimum and maximum values. All results include 3 biological replicates. Ab/iso, ratio of Ab MFI over isotype control MFI.****

BAL27862 has a cytostatic and cytolytic effect on SB28 cells (Supplemental Figure 1; supplemental material available online with this article; https://doi.org/10.1172/jci.insight.142980DS1), with an IC_50_ of 5.5 nM (Figure 1A). We also assessed BAL27862’s induction of key molecules associated with immunogenic cell death (ICD), namely, extracellular release of ATP and high mobility group box 1 (HMGB1), and translocation of calreticulin to the membrane. BAL27862 did not induce extracellular release of ATP or calreticulin translocation (Figure 1, B and C). In contrast, the compound modestly increased extracellular release of HMGB1 at all concentrations tested (Figure 1D). These results indicate that SB28 is sensitive to BAL27862 at in vivo–achievable concentrations and that the resulting dying cells might stimulate recognition by innate immune cells through release of HMGB1.

### BAL101553 prolongs survival of mice bearing orthotopic, TMZ-resistant SB28 glioma but does not sensitize to ICB.

**Figure 2:**
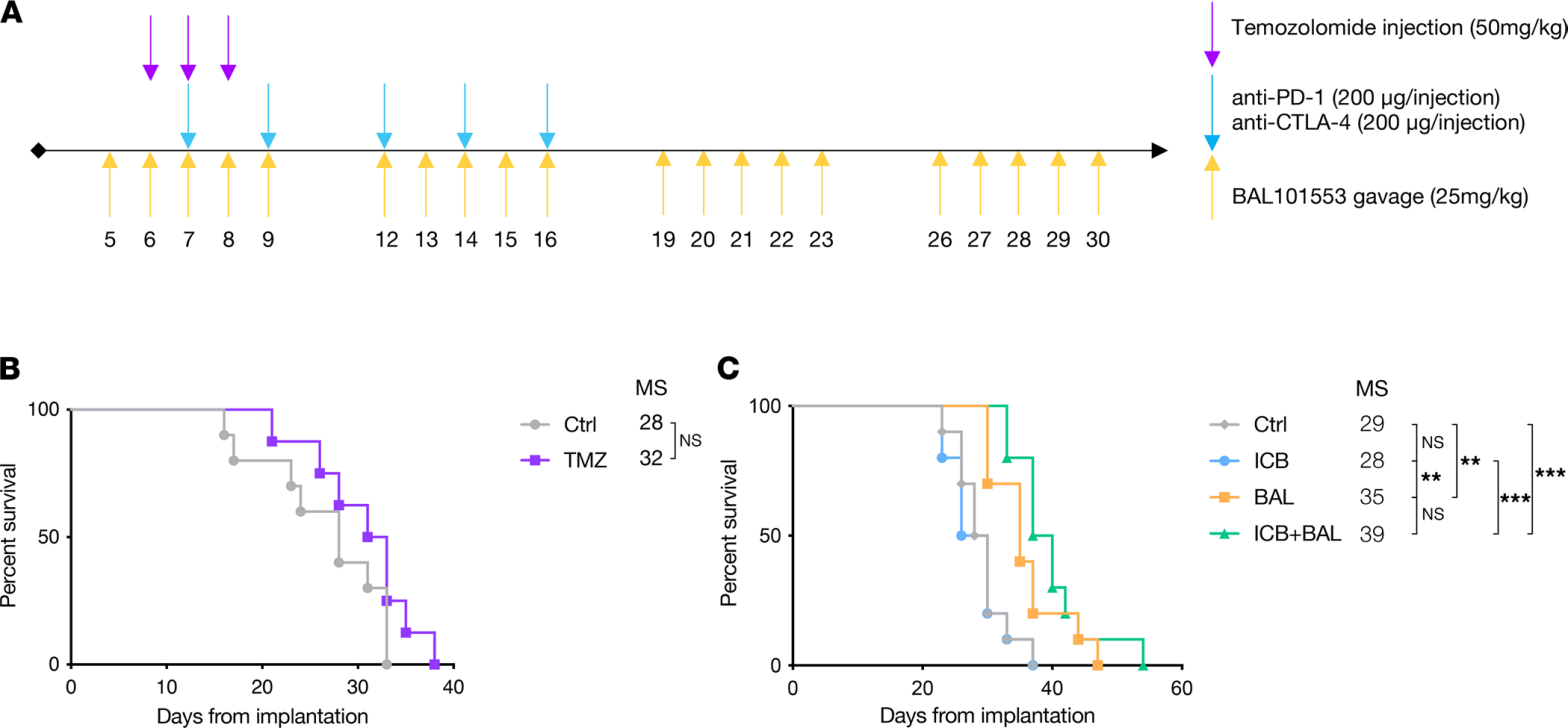
BAL101553 but not TMZ significantly improves survival of SB28-implanted mice. (**A**) Treatment schedule of mice IC implanted with SB28 at day 0. (**B**) Symptom-free survival curve of mice treated with temozolomide (TMZ) or vehicle control (Ctrl). (**C**) Symptom-free survival curve of mice treated with anti–PD-1 and anti–CTLA-4 (ICB), BAL101553 (BAL), or a combination of both treatments (ICB+BAL). Treatments were injected intraperitoneally except for BAL101553, which was administered by oral gavage. Median survival (MS) is displayed in days for survival curves. Statistics: Log-rank (Mantel-Cox): nonsignificant: *P* > 0.05; **: *P* < 0.01; ***: *P* < 0.001. *n* = 10 mice per group.

We assessed the efficacy of TMZ treatment in mice implanted intracranially (IC) with SB28 (Figure 2A), a protocol previously reported to be efficacious in the GL261 model (22). SB28-implanted mice were resistant to TMZ therapy, with no increase in survival (Figure 2B). We then proceeded to in vivo testing with the prodrug BAL101553 to determine whether the in vitro benefits of BAL27862 could be recapitulated in vivo. BAL101553 monotherapy resulted in prolonged survival of implanted mice that was statistically significant (Figure 2C), resulting from delayed SB28 growth assessed by in vivo bioluminescence imaging (Supplemental Figure 2). However, once tumor growth was unequivocal, growth rate was similar in control and BAL101553-treated mice. Histological analysis of brains at an intermediate time point (21 days postimplantation) also showed a clear reduction in tumor size in BAL101553-treated mice compared with controls (Supplemental Figure 3). To further optimize BAL101553 effect, we tested treatment durations of 2 and 4 weeks, or until appearance of terminal symptoms. The latter treatment duration significantly increased survival compared with 2 weeks (42 vs. 35 days of MS) but not with 4 weeks of treatment (Supplemental Figure 4, A and B).

We next evaluated the effects of combining BAL101553 treatment with ICB (consisting of a combination of aPD-1 and aCTLA-4) using an established protocol previously shown to be efficacious in the GL261 mouse glioma model but not in SB28 (15). We confirmed resistance of SB28-implanted mice to ICB alone, as no survival increase was observed. Moreover, combination of BAL101553 with ICB did not reveal any statistically significant impact over BAL101553 treatment alone (MS: 35 days vs. 39 days for BAL101553 or BAL101553 and ICB, respectively) (Figure 2C).

BAL101553 is an activator of the SAC, inducing cell cycle arrest and subsequent death, or aberrant chromosome segregation leading to genomic instability (23). This in turn could induce mutations and promote neoepitope generation, thereby potentiating the ICB effect. We hypothesized that late combination with ICB, when BAL101553 had already affected tumor cells for an extended time period, might be most efficacious. Moreover, we reasoned that even low doses of BAL101553, not leading to immediate cell death, could also efficiently drive genomic instability. We therefore tested late combination of ICB and a lower dose (15 mg/kg) of BAL101553. However, late use of ICB, either alone or after extended BAL101553 treatment, did not reveal any ICB/BAL101553 combination effect (Supplemental Figure 4C). Regarding low-dose treatment with BAL101553 at 15 mg/kg, this was as potent as 25 mg/kg to prolong survival: 40 and 42 days of MS for 15 mg/kg and 25 mg/kg, respectively, compared with 29 days of MS for controls. However, the lower dose of BAL101553 still did not induce ICB responsiveness (Supplemental Figure 4D).

### SB28 cells acquire an IFN-γ response signature in vivo.

**Figure 3:**
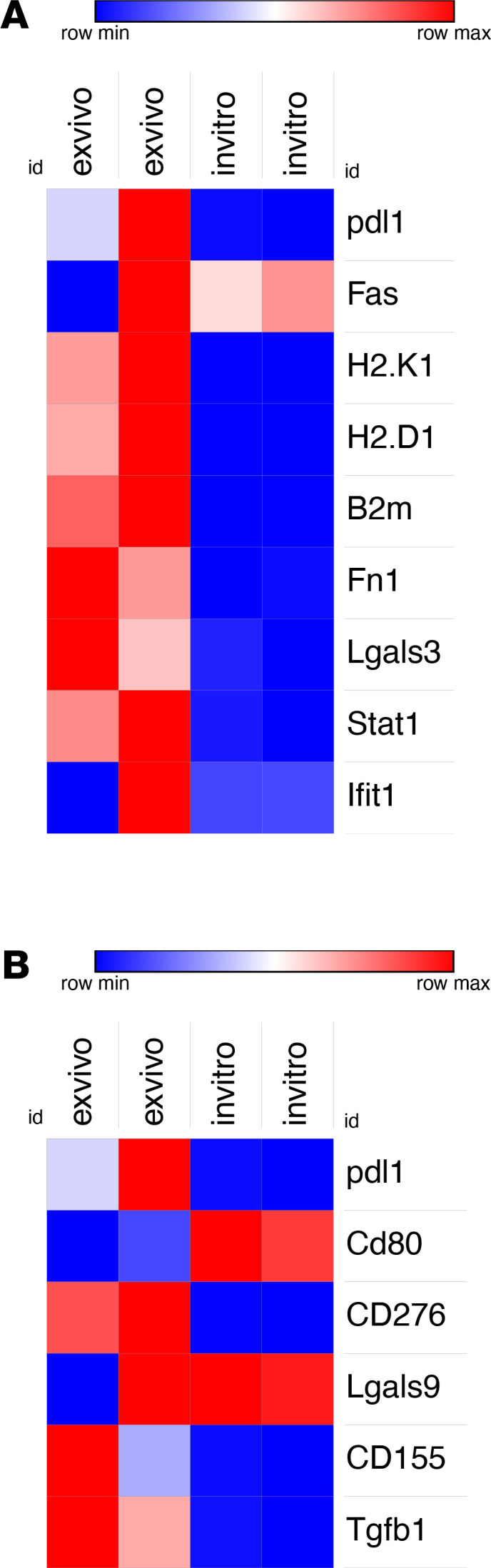
IFN-γ signature and immunomodulatory molecule gene expression by SB28 glioma cells after in vivo growth. (**A** and **B**) Gene expression level of genes comprising (**A**) an IFN-γ signature and (**B**) key immunomodulatory molecules, based on RNA sequencing between in vitro and ex vivo SB28 cells, respectively. Two biological replicates are analyzed for each group.

RNA sequencing was used to assess transcriptional changes in flow cytometry–sorted SB28 cells resulting from 21 days or 33 days of in vivo growth (mice treated with vehicle control or BAL101553, respectively). There was a prominent upregulation of expression of IFN-γ response genes in ex vivo samples compared with their in vitro counterparts (Figure 3A). Expression of *B2m*, *H2.D1*, and *H2.K1*, coding for MHC-I chains, was significantly upregulated. Other IFN-γ–regulated genes, such as *Fn1*, *Lgals3*, and *Stat1*, were also expressed at a significantly higher level by ex vivo samples (Figure 3A). Interestingly, *Pdl1*, which is included in the IFN-γ signature, was significantly upregulated, as well as other genes encoding (potentially) immunoregulatory molecules such as *CD80*, *CD155*, *Tgfb1*, and *CD276* (B7H3) but that are not part of the IFN-γ signature (Figure 3B). In order to validate the presence of IFN-γ cytokine that could induce this pattern of gene expression by the tumor cells, we tested whole tumor lysates with an IFN-γ ELISA (data not shown); this showed a trend for more IFN-γ in tumor-bearing mice compared with healthy brains, presumably derived from infiltrating T cells and NK cells. In contrast to the in vitro and ex vivo differences in gene expression by SB28 cells from nontreated mice, no significant differences were observed between ex vivo controls and SB28 cells sorted from BAL101553-treated mice, neither for the IFN-γ signature nor for immunoregulatory molecule expression (Supplemental Figure 5, A and B).

### BAL101553 synergistically combines with aCD40 to increase survival.

**Figure 4:**
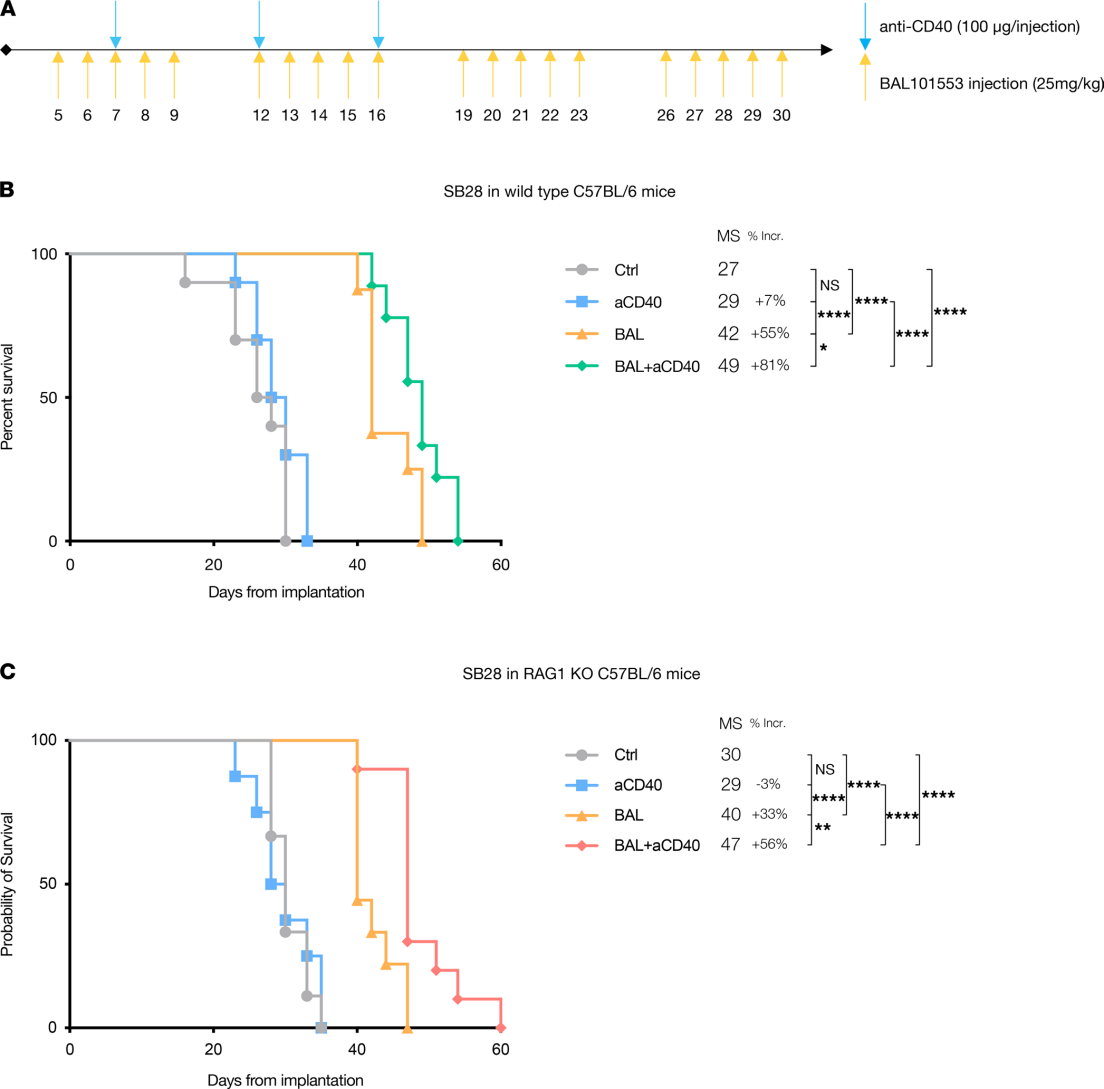
Early anti-CD40 combination with BAL101553 therapy offers a significant survival increase in SB28-implanted T/B cell–deficient mice. (**A**) Treatment schedule of mice IC implanted with SB28 at day 0. (**B** and **C**) Symptom-free survival after treatment with anti-CD40 (aCD40), BAL101553 (BAL), or a combination of both (BAL+aCD40) of (**B**) wild-type, immunocompetent C57BL/6 mice or (**C**) immunodeficient B6.129S7-Rag1^tm1Mom^/J (RAG1 KO) mice. MS is indicated in days, and the percentage increase (% Incr.) in MS is calculated relative to Ctrl group. Statistics: Log-rank (Mantel-Cox): nonsignificant *P* > 0.05; *: *P* < 0.05; **: *P* < 0.01; ****: *P* < 0.0001. *n* = 8–10 mice per group.

Because SB28 is an immunologically cold tumor (15), and because BAL101553 therapy cannot potentiate an ICB effect in 2 combination treatment protocols, we hypothesized that immunogenicity of SB28 might be augmented by immune activation in addition to ICB. We tested an agonistic aCD40 antibody, reasoning that this may promote macrophage and dendritic cell (DC) activation, which in turn would promote T cell–dependent antitumor immunity. We first tested a late combination with ICB, in order to have the tumor affected by BAL101553 treatment, and aCD40 used concomitantly with ICB (Supplemental Figure 6A). Adding agonistic aCD40 alone did not prolong survival (Supplemental Figure 6B); all survival-extending treatments required BAL101553, either alone or in combination with aCD40 or with ICB. The modest, but nonsignificant, trend for prolonged survival with the BAL101553 and aCD40 combination encouraged us to try to enhance the effect and to explore potential mechanisms. Because we had determined that prolonged exposure to BAL101553 did not significantly increase mutational load (Supplemental Figure 7), there was less rationale for late combination of immunotherapy. We hypothesized that combining aCD40 from day 7 postimplantation (as in Figure 4A) might enhance its combinatory effect. Indeed, there was a modest but significant increase of survival when BAL101553 was combined with aCD40, compared with BAL101553 monotherapy (MS of 42 days and 49 days in BAL101553 and BAL101553 plus aCD40 groups, respectively; Figure 4B). This combination treatment resulted in an 81% increase in survival compared with control mice (Figure 4B). Notably, aCD40 monotherapy was inefficacious, with combination therapy needed to reveal a synergistic treatment effect.

### Efficacy of BAL101553 and aCD40 combination therapy is T cell independent and affects BM-derived macrophage tumor infiltration.

To identify immune subsets modulated by the partially efficacious BAL101553 plus aCD40 combination therapy, we analyzed brain-infiltrating lymphocyte (BILs) at different time points. The immune infiltrate was significantly affected by the different treatments, in particular with aCD40 monotherapy (Supplemental Figure 8), with putative protumor (shown in red) or antitumor (shown in green) immune subsets indicated for the different treatment protocols. Complete analyses of mice sacrificed at appearance of terminal symptoms showed a significant increase in infiltrating T cells, with a phenotype compatible with fewer exhausted (PD-1^+^ and killer cell lectin like receptor G1 negative, KLRG1^–^) but more memory (PD-1^–^KLRG1^+^) CD8^+^ T cells and a higher CD8/CD4 and CD8/Treg ratio in the aCD40-treated group (Supplemental Figure 8, G–I) (24). BAL101553 monotherapy also affected BILs, with higher MHC-II expression by DCs, and more NK cell infiltration at day 33 after tumor implantation (Supplemental Figure 8, J and K), but this effect was lost at the time of terminal symptoms. Notably, upon appearance of terminal symptoms, mice treated with BAL101553, as monotherapy or in combination with aCD40, showed lower macrophage expression of PD-L1 compared with controls or aCD40-treated mice (Supplemental Figure 8O).

Overall, considering flow cytometry data and immunohistology (data not shown), no single innate or adaptive immune subset could be directly correlated with the synergistic effect of BAL101553 and aCD40. To gain insight into roles of innate versus adaptive immune cells, we repeated the same treatment regimens with tumor-bearing B6.129S7-Rag1^tm1Mom^/J mice, lacking T and B lymphocytes. The survival advantage offered by aCD40 monotherapy and the synergistic effect of BAL101553 and aCD40 seen in immunocompetent mice were fully recapitulated in these T cell– and B cell–immunodeficient mice (Figure 4C). These results unequivocally exclude T cells (or B cells) as being involved in the therapeutic effect of this antineoplastic and immunomodulatory combination treatment and instead implicate innate immune cells, present in both mouse strains.

**Figure 5:**
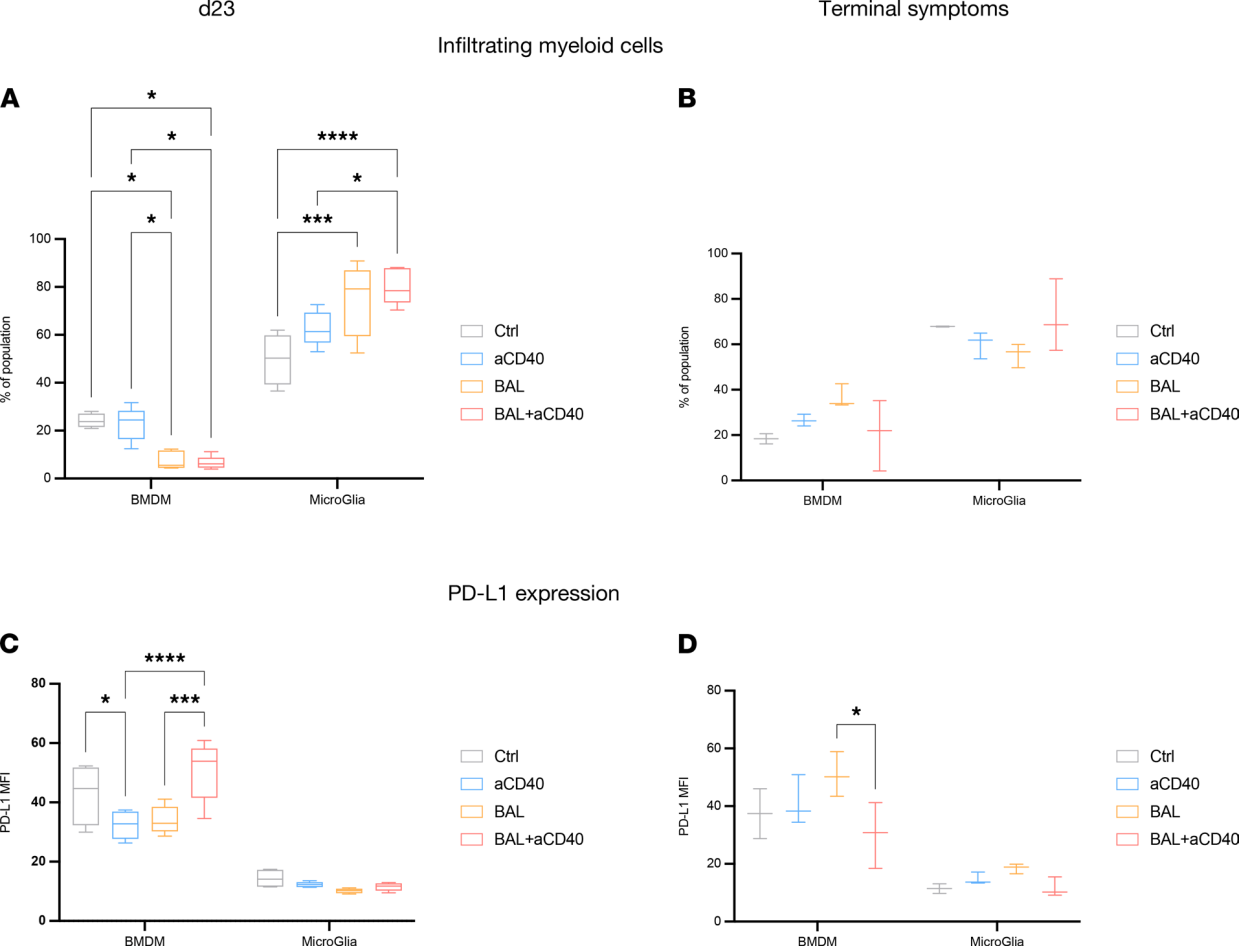
BAL101553-treated tumors are less infiltrated by BM-derived macrophages. (**A** and **B**) Proportion of brain-infiltrating myeloid cells, and their corresponding PD-L1 expression (**C** and **D**), from mice IC implanted with SB28 and treated with vehicle control (Ctrl), anti-CD40 (aCD40), BAL101553 (BAL), or a combination of BAL and aCD40 were collected at day 23 (**A** and **C**) or at time of terminal symptoms (**B** and **D**). Statistics: 2-way ANOVA with Tukey’s multiple-comparison test. *: *P* < 0.05; ***: *P* < 0.001; ****: *P* < 0.0001. Error bars indicate SD. BMDM cells are defined as CD11b^+^CD49d^+^, microglia as CD11b^+^CD49d^–^. All cells are gated on CD45^+^ cells after exclusion of doublets and dead cells. Box-and-whisker plot with the bounds of the box representing lower and upper quartiles, the line within the box showing the median, and the whiskers showing minimum and maximum values. *n* = 3–4 mice per group.

Considering the T cell–independent therapeutic effect of BAL101553 and aCD40 combination, we conducted more detailed analysis of myeloid cells’ infiltration. In Figure 5, we see significantly less BM-derived macrophage (BMDM) tumor infiltration compared with microglia cells at day 23 after tumor implantation. Moreover, PD-L1 expression of BMDM was significantly enhanced after combination therapy when compared with single modality treatments. DC subsets were also characterized, with higher MHC-II expression (Supplemental Figure 8J) but lower cDC2 (CD45^+^CD11c^+^CD8^–^) infiltration in mice treated with BAL101553 (Supplemental Figure 9).

### Combination of BAL101553 and aCD40 is also efficacious in GL261-implanted mice.

**Figure 6:**
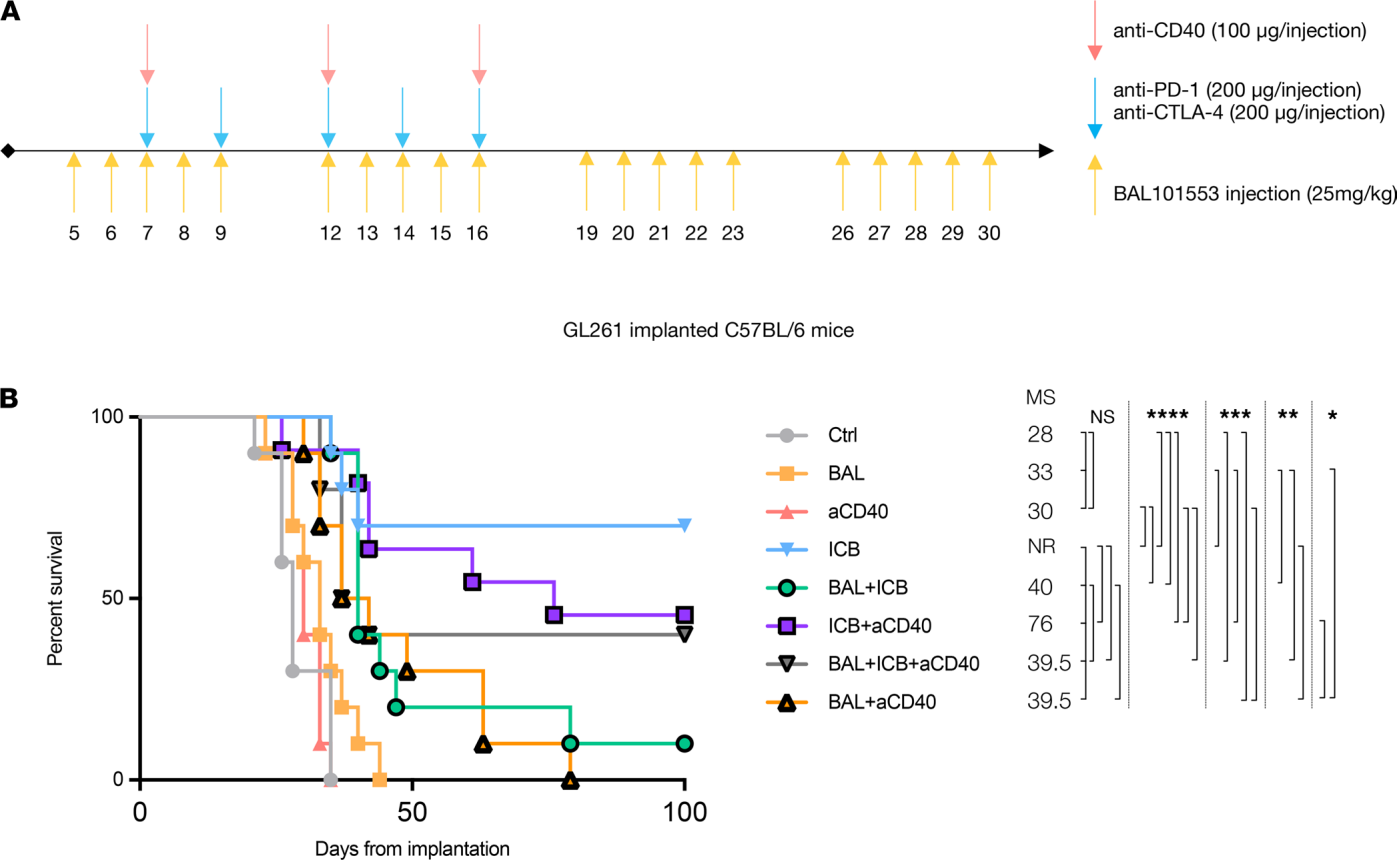
Combination of BAL101553 and aCD40 significantly improves survival of GL261-implanted mice. (**A**) Treatment schedule of mice IC implanted with GL261 at day 0. (**B**) Symptom-free survival of mice treated with vehicle control (Ctrl), anti-CD40 (aCD40), BAL101553 (BAL), immune checkpoint blockade (ICB), a combination of BAL+ICB, a combination of BAL+aCD40, a combination of ICB+aCD40, and a combination of BAL+ICB+aCD40. MS is displayed in days. Statistics: Log-rank (Mantel-Cox): nonsignificant *P* > 0.05; *: *P* < 0.05; **: *P* < 0.01; ***: *P* < 0.001; ****: *P* < 0.001. *n* = 10 mice per group.

In order to assess the potential of therapeutic combinations of ICB and aCD40 with BAL101553 in more highly infiltrated and mutated GBM, we also tested treatment combinations in GL261-bearing mice (15) (Figure 6A). GL261 cells were less sensitive than SB28 to BAL27862 in vitro, with an IC_50_ of 13.4 nM (Supplemental Figure 10). Consistent with this, BAL101553 monotherapy, using the same regime that was efficacious in SB28-bearing mice, did not affect survival in the GL261 model (Figure 6B). However, when BAL101553 was combined with aCD40 (inefficacious as monotherapy), there was a significant survival benefit, although all mice eventually succumbed to tumor growth. This contrasts with the efficacy of ICB therapy in this model, which, as expected in this highly mutated tumor (15, 25), led to long-term survival in 7/10 mice. Surprisingly, combination of BAL101553 and ICB had a significantly lower effect than ICB alone, with only 1/10 mice surviving at the end of the follow-up (100 days after tumor implantation) (Figure 6B).

## Discussion

There is a major unmet clinical need to develop new treatments for GBM, but immunotherapy is proving challenging to use effectively. Indeed, a phase III clinical trial (26) showed no benefits from aPD-1 therapy compared with bevacizumab, although use of nivolumab in a neoadjuvant setting was more encouraging (27, 28). Nevertheless, more widespread application of immunotherapies for GBM will require stringent preclinical testing using models that recapitulate both ICB-responsive tumors and ICB-resistant tumors, exemplified by the GL261 and SB28 models previously described (15) and further investigated in the present study. Our results show that even in the poorly mutated and immunogenic SB28 GBM model, there is still evidence for immune activation, with IFN-γ measured in the tumor (data not shown) and an IFN-γ response gene signature detected ex vivo. Moreover, PD-L1 was upregulated in SB28 tumors after in vivo growth, validating the rationale to use an ICB strategy. However, use of aPD-1 and aCTLA-4 ICB in the context of SB28 treatment never improved survival in our experiments, indicating that further treatment combinations need to be considered. Combination therapies have been successfully used to treat an ICB-resistant mouse pancreatic cancer model, using radiation, antineoplastic agents, and immunomodulators (12), or with a hypoxia-activated prodrug together with double ICB in transgenic prostate models (29). Even the highly resistant SB28 GBM model showed some responsiveness to a triple therapy comprising TMZ, a Na/H exchanger blocker, and aPD-1, although there were no long-term survivors (30). The number of possible treatment protocols when 2, 3, or more compounds or modalities are combined will rapidly exceed what is testable clinically, and so maximum insight must be gained from preclinical investigation (31). Moreover, using more than 1 model is likely to be helpful in order to recapitulate subgroups of patients.

In human GBM, patients with a hypomethylated O^6^-methylguanine–DNA methyltransferase do not benefit from the addition of TMZ to radiotherapy (32). Few alternative chemotherapy agents efficiently reach the tumor site in the brain, hence the interest in BAL101553, which efficiently enters the brain of treated rodents (20), is active in human GBM models (21, 33), and is currently undergoing clinical evaluation in GBM patients (ClinicalTrials.gov identifiers NCT03250299 and NCT02490800). In our experiments, SB28 GBM was also insensitive to TMZ, but responsive to BAL101553 treatment, in vitro and in vivo, with marked survival increase but no long-term survivors. The initial slower growth rate of SB28 tumors in BAL101553-treated mice (as judged by bioluminescence) would be compatible with a cytostatic action of the compound, which we did observe in vitro, in addition to cytotoxicity. However, the subsequent growth rate was similar in treated and untreated mice, which would be compatible either with an acquired resistance mechanism and outgrowth of resistant cells or with decreased drug availability to some tumor cells after a certain volume increase. Antineoplastic agents can potentially synergize with immunotherapy if it stimulates innate immunity through ICD (34) or adaptive immunity through enhanced neoepitope expression. BAL101553 did not strongly induce ICD in SB28 cells, although there was a modest increase in the release of HMGB1, which has been reported to bind to TLR2 and TLR4 and to enhance GBM infiltration by DCs (35). Consistent with this, we observed higher activation of brain-infiltrating DCs in BAL101553-treated, SB28-bearing mice. Some antineoplastic drugs can induce mutations even at subcytotoxic doses, leading to neoantigen expression, which may augment tumor immunogenicity. We tested multiple treatment protocols to optimize SB28 exposure to BAL101553, to induce mutations and thereby favor ICB responsiveness. However, the time period over which the compound can act on tumor cells in a mouse model is shorter than would occur in human patients. Consequently, we observed no significant increase in mutational load after treatment, although the number of predicted neoepitopes was slightly higher (Supplemental Figure 7D). Nevertheless, these approaches did not sensitize to ICB. In human GBM, some cases of hypermutated GBM are described, particularly at recurrence after TMZ treatment (36), but their sensitivity to ICB seems limited (37). In contrast, encouraging clinical responses are observed after neoadjuvant ICB in recurrent GBM patients (27, 38) that may reflect higher mutational load after prior TMZ treatment, although this potential mutational load increase was not reported.

Although ICB is currently championed as the major breakthrough in cancer immunotherapy, considerable fundamental, preclinical, and clinical knowledge in immune stimulation also exists, such as by vaccination, cytokines, or the use of agonistic antibodies (39). Clinical success of such approaches as monotherapies has been limited, but now with so many validated therapeutics available for ICB and for immunostimulation, combining the 2 approaches for the most refractory cancers is a compelling way forward (40). Among the possibilities, agonistic aCD40 is particularly interesting because it can work at many levels, for example, by activating DCs to more efficiently stimulate T cells, by triggering macrophages to exhibit antitumor functions, or even by directly interacting with certain CD40-expressing tumor cells, as proposed for certain human gliomas (41, 42), although this would not be the case for the CD40-negative mouse models used here (15). Use of aCD40 monotherapy for the immunologically cold SB28 GBM was not surprisingly inefficacious and indeed was previously reported (43). Nevertheless, aCD40 did favorably modulate infiltration of some immune cell subsets, including increased T cell infiltration. More interestingly, combining aCD40 with BAL101553 synergistically improved survival, but further improvement was not attained by adding ICB. It is possible that neoantigen expression was still insufficient to be targeted by antitumor T cells, as proposed in pancreatic cancer, a malignancy with similarities to GBM, such as resistance to ICB and low mutational load (44). Finally, since aCD40 and BAL101553 treatment of SB28 was equally efficacious in T cell– and B cell–deficient mice, this excludes a role of adaptive immunity, suggesting that SB28 GBM progresses without T cell–mediated immunoediting and is intrinsically resistant to killing by endogenous T cells. Such tumors might be better targeted by transfer of engineered T cells if suitable antigens could be targeted (45).

Despite the lack of involvement of T cells in the therapeutic response of SB28 to aCD40 and BAL101553, the fact that a robust survival increase was observed highlights the interest of exploiting innate immunity in antineoplastic/immunotherapeutic combinations for poorly antigenic/immunogenic tumors. Myeloid cells, as the major CD40-expressing immune cell in the GBM microenvironment (in humans and also in the SB28 and GL261 mouse models), merit further investigation as a target of immunomodulation. Their origin and phenotype can significantly impact their function, with M0 and M2 polarized macrophages being described in GBM (11, 46) and a CD73^hi^ macrophage signature being associated with poor prognosis (31). Our combination therapy decreased BMDM infiltration compared with microglia, and impacted PD-L1 expression, suggesting a protumor role of BMDMs and that a reduction in their infiltration favors survival. Moreover, in the GL261 model, PD-L1^+^ macrophages were proposed to be responsible for ICB resistance in the proportion of mice unresponsive to treatment (47). Inhibition of colony stimulating factor 1 receptor (CSF1R) has been tested to modulate GBM-associated macrophages, but resistance or clinical unresponsiveness was reported (11, 48). Combination therapies will need to be considered, which might also reinvigorate antitumor T cells (if present), as shown for a combination of aCD40 and CSF1R inhibitors (49). A further innate immune cell that should be considered is the NK cell, which we observed to be transiently augmented after BAL101553 treatment. The low or absent MHC expression by SB28 (15) would favor NK interaction with GBM cells, although this situation would likely be reversed after NK secretion of IFN-γ, supported by our observation of the IFN-γ response signature we detected ex vivo from SB28-bearing mice. As aCD40 and BAL101553 therapy showed some impact on DCs, this could also favor DC-NK crosstalk (50) in the SB28 model in which the role of T cells appeared with be minimal. The therapeutic interest of NK cells is considerable, particularly in view of their putative roles as effector cells able to kill human stem-like GBM-initiating cells (51).

Interestingly, the combination of BAL101553 and aCD40 was also efficacious in the well-infiltrated GL261 model, even though these agents were ineffective as monotherapies. However, in view of the characteristics of the GL261 tumor cells, particularly high mutational load, high MHC-I expression (15), and an intense immune infiltrate, GL261 probably only models a small number of human GBM cases. Unsurprisingly, our results from this study, and those from several other groups, suggest that successful treatments for the GL261 model will be T cell mediated (13, 52). Notably, the treatment combination of ICB together with BAL101553 was less efficacious than ICB alone, which might represent supraoptimal modulation of the TME or some cytotoxic/cytostatic action of BAL101553 on T cells during their activation, which we observed in vitro using BAL27862 (data not shown). This situation is unlikely to be encountered in clinical immunotherapy for GBM, particularly if the ICB approach does not include CTLA-4 antagonism. In conclusion, we have identified treatment combinations for GBM models that exhibit different responsiveness to the mitotic spindle checkpoint controller BAL101553 or immunomodulators (ICB and aCD40). Poorly immunogenic SB28 GBM, with a low mutational load, was modestly responsive to BAL101553 as monotherapy, and survival was further enhanced by addition of aCD40 stimulation, a treatment that functioned independently of T cells but impacted BMDMs. The more immunogenic and highly mutated GL261 responded to ICB alone (aPD-1 and aCTLA4), but the only therapeutically advantageous combination therapy was BAL101553 and agonistic aCD40. Our results highlight that the immune characteristics of the tumor may override the cancer cell of origin in predicting therapy response treatment combinations, a factor that should be considered for selecting patients for testing future therapy combinations in GBM.

## Methods

### Cell lines.

SB28 and GL261 murine cell lines (provided by H. Okada, UCSF, San Francisco, California, USA) were tested as mycoplasma negative and were cultured as previously described (15). SB28 expresses GFP and luciferase, used for cell sorting, immunofluorescence, and in vivo bioluminescence monitoring.

### In vitro tests on SB28 cells.

SB28 cells were seeded in 96-well plates for 24 hours, then further incubated for 48 hours with the indicated concentrations of BAL27862 (provided by Basilea Pharmaceutica International Ltd., Basel, Switzerland) of 0–60 nM, or the corresponding solvent concentration (DMSO). The median 50% growth inhibition of BAL27862 across a panel of tumor cell lines is 12 nM, a concentration readily achievable in mouse plasma based on the average serum concentration (AUC_0–24h_/24) following a dose of 20 mg/kg, orally (data not shown). CellTiter-Glo Luminescent Cell Viability/Proliferation kit (Promega) was used to assess viable cell number based on total ATP content. Culture supernatants were assessed for extracellular ATP and HMGB1 release by an ENLITEN ATP Assay (Promega) and an HMGB1 ELISA (Tecan), respectively. Cell surface calreticulin antibody was assessed by flow cytometry on detached cells using ab2907 antibody (Abcam).

### BILs’ isolation and flow cytometry analysis.

Mice were sacrificed, then perfused transcardially with Ringer’s solution, and BILs were partially purified as described previously (53). Cells were incubated with LIVE/DEAD Fixable Dead Cell Stain Kit (Invitrogen, Thermo Fisher Scientific), blocked for Fc receptor binding with 2.4G2, then surface stained with the following Ab: CD45 (30-F11), CD11b (M1/70), CD49d (R1-2), CD62L (Mel14), Ly6G (1A8), Ly6C (HK1.4), CD103 (M290), CD8 (53–6.7), CD4 (GK1.5), PD-1 (29F.1A12), PD-L1 (MIH5), NK1.1 (PK136), MHC-II (I-A/I-E, M5/114.15.2), CD80 (16-10A1), CD86 (GL-1), CD11c (N418), CD3 (145-2C11) and KLRG1 (2F1/KLRG1). For intracellular staining, we used Cytofix/Cytoperm kit (BD Biosciences) and stained for FoxP3 (FJK-16s, BD Biosciences). All Ab were purchased from BioLegend, except CD103 (M290) and CD8 (53-6.7) from BD Biosciences. Stained cells were analyzed on a Gallios flow cytometer (Beckman Coulter) and data analyzed using Kaluza software (Beckman Coulter).

### Proliferation and apoptosis assay on SB28 cells.

SB28 cells were incubated with CellTrace Violet (Invitrogen, Thermo Fisher Scientific) and seeded in 12-well plates for 24 hours. They were then further incubated for 48 hours with the indicated concentrations of BAL27862 (provided by Basilea Pharmaceutica International Ltd., Basel, Switzerland) or the corresponding solvent concentration (DMSO). Cells were incubated with LIVE/DEAD Fixable Dead Cell Stain Kit (Invitrogen, Thermo Fisher Scientific) and annexin V PE conjugated (Immunotools). Stained cells were analyzed on a Gallios flow cytometer and data analyzed using Kaluza software.

### Mouse implantation, bioluminescence, and in vivo treatment.

Tumor cells were implanted orthotopically as previously described (15), using 6- to 8-week-old C57BL/6J mice (Charles River Laboratories) or B6.129S7-Rag1^tm1Mom^/J mice (The Jackson Laboratory). Mice were monitored daily for weight loss or symptom appearance and euthanized according to veterinarian-authorized endpoints. For bioluminescence monitoring, mice were injected intraperitoneally (IP) with 3 mg of Luciferin (Gold Biotechnology) 5 minutes prior to image acquisition on an IVIS Spectrum system (Xenogen, PerkinElmer) with 3 images collected at 5-minute intervals. Only the highest signal was used for quantification. Therapeutic Ab were all purchased from Bio X Cell: anti–PD-1 (RMP1-14), anti–CTLA-4 (9D9), anti-CD40 (FGK4.5), rat IgG2a isotype control (2A3), and mouse IgG2b isotype control (MPC-11) were injected IP as specified. The prodrug BAL101553 was provided by Basilea Pharmaceutica International Ltd., administered by oral gavage at the indicated concentrations. TMZ was purchased from MilliporeSigma and injected IP at 50 mg/kg dose.

### Ex vivo cell sorting.

At day 21 or 35 after tumor implantation, for controls or BAL101553-treated mice, respectively, to match for tumor volume, mice were sacrificed and brains dissected. Tumor cells were obtained using a Brain Dissociation Kit (Miltenyi Biotec), staining with anti-CD45 (30-F11) and anti-CD11b (M1/70) Ab from BioLegend, then sorting on a FACSAria II (Becton Dickinson), with gating on CD45^–^CD11b^–^GFP^+^ cells. We collected a total of 300,000 cells per sample.

### Sequencing data and analysis.

We extracted DNA and RNA from the sorted samples using an AllPrep DNA/RNA Mini Kit (QIAGEN). Agilent protocol with 100 paired-end reads was used for whole-exome sequencing. To analyze the data, we used the following programs: MuTect or Haplotype Caller for variant calling and SNPeff for variant annotation. We report single nucleotide variant and frameshift mutations for each sample. NetMHCPan 4.0 or NetMHCIIPan 4.0 and IEDB v2.18 were used to predict neoepitope binding to MHC-I and -II. TruSeq stranded RNA protocol on Illumina was used for RNA sequencing, and the 50 bp reads were mapped with TopHat v2.3.13. PicardTools v1.80 was used for quality control and summarization. HTSeq and Rsubread featurCounts obtained the counts. Differential expression analysis after normalization was performed by R/Bioconductor package edgeR v3.4.3. For IFN-γ signature and differential expression of immunoregulatory molecules, we performed waterfall analysis using R. We sequenced 2 ex vivo samples for each control mice or BAL101553 group and report the results as median of both samples or a combination of all genes expressed in the 2 samples. WES and RNA sequence data are available on National Center for Biotechnology Information’s Gene Expression Omnibus, accession number: GSE127075.

### Histology and immunofluorescence.

Brains were collected after transcardiac perfusion, then transferred to a solution of 30% sucrose overnight. Tissues were then embedded in Tissue-Tek OCT (Sakura), frozen with liquid nitrogen, and stored at –80°C. We used a Leica cryostat for sectioning. Sections were fixed with 4% paraformaldehyde and blocked with a solution of 2.5% goat serum and 5% BSA (MilliporeSigma). The following primary Ab were used: rabbit anti-GFP (Proteintech, 50430-2-AP), rat anti-CD31 (BioLegend, MEC13.3), rat anti-F4/80 (BD Biosciences, T45-2342), rat anti-Ki67 (BD Biosciences, B56), and rat anti-NK1.1 (BioLegend, PK136). For the secondary Abs, we used goat anti-rabbit AF488 (Abcam, ab150077) and mouse anti-rat eFluor 660 (eBioscience, Thermo Fisher Scientific, r2a-21B2). Image acquisition was performed on a Zeiss Axio Imager Z1, Axio Imager.Z2 Basis LSM 800, or Zeiss Axioscan.Z1 microscope with either ×20 or ×40 objectives and processed using Zeiss Zen software.

### Statistics.

For survival curves we used log-rank (Mantel-Cox), and for multiple comparisons (Figure 1, B–D; and Figure 5, A–D), we used 2-way ANOVA with Tukey’s multiple-comparison test. *P* < 0.05 was considered significant.

### Study approval.

All animal experimental studies were reviewed and approved by institutional — Direction de l’expérimentation animale, Geneva, Switzerland — and cantonal — Direction générale de la santé, Geneva, Switzerland — veterinary authorities in accordance with Swiss Federal law.

## Author contributions

VG and PRW conceived and designed the experiments. VG, FIE, and VR performed the experiments. VG, FIE, EM, VR, PYD, PM, FB, HAL, and PRW analyzed the data. VG, FIE, EM, VR, PYD, PM, FB, HAL, and PRW wrote the paper.

## Supplementary Material

Supplemental data

## Version 1 08/17/2021

In-Press Preview

## Version 2 09/22/2021

Electronic publication
