# Supplementary material for: Treating ICB-resistant glioma with anti-CD40 and mitotic spindle checkpoint controller BAL101553 (lisavanbulin)
Source: JCI Insight. 2021 Sep 22;6(18):e142980. doi: 10.1172/jci.insight.142980 (PMC8492343; doi:10.1172/jci.insight.142980)
Supplement: Supplemental data [file jciinsight-6-142980-s039.pdf]

Supplementary Figure S1

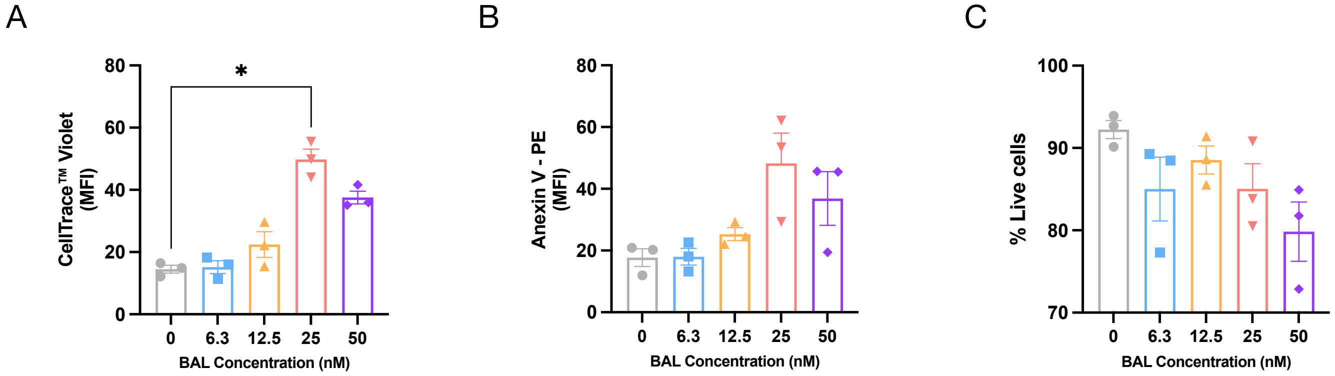

Supplementary Figure S1 – In vitro, BAL27862 has a cytostatic and cytolytic activity on SB28 cells. SB28 cells were exposed in vitro to the indicated concentrations of BAL27862 for 48 hours and analyzed by flow cytometry for proliferation (A), apoptosis (B) and cell death (C). Mean +/- SEM indicated of 3 independent experiments. Statistics: Kruskal-Wallis test: *: $p<0.05$.

Supplementary Figure S2

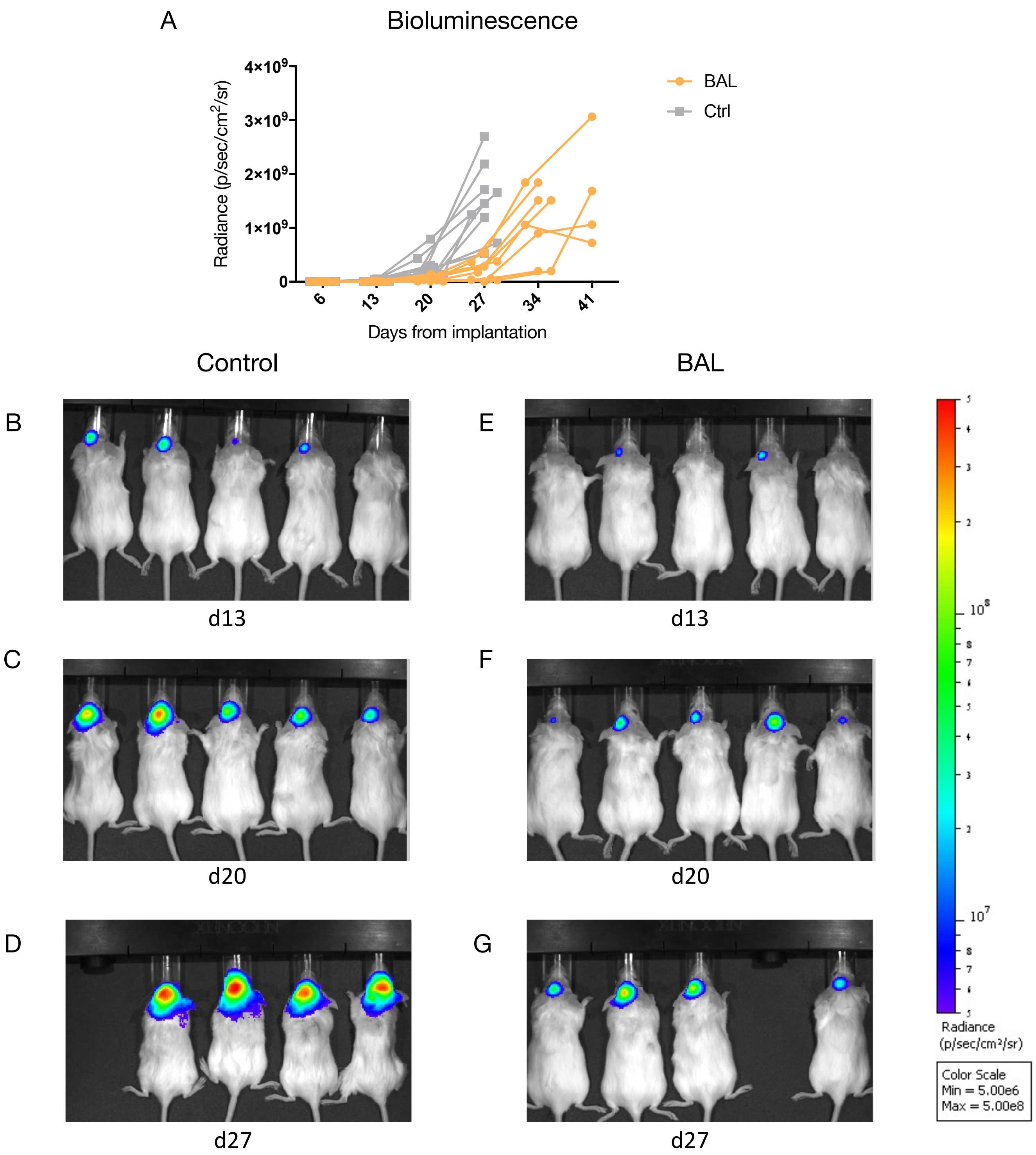

Supplementary Figure S2 – BAL101553 delays SB28 growth in vivo as shown by bioluminescence follow-up. (A) Individual bioluminescence signal follow-up of all mice intra-cranially implanted with SB28 and treated with BAL101553 (BAL) or vehicle control (Ctrl) until terminal symptoms. (B, C, D, E, F, G) Representative pictures of bioluminescence follow-up at day 13 (B, E), 20 (C, F) or 27 (D, G) post intra-cranial implantation of SB28, of mice treated with vehicle control (B, C, D) or BAL101553 (E, F, G).

Supplementary Figure S3

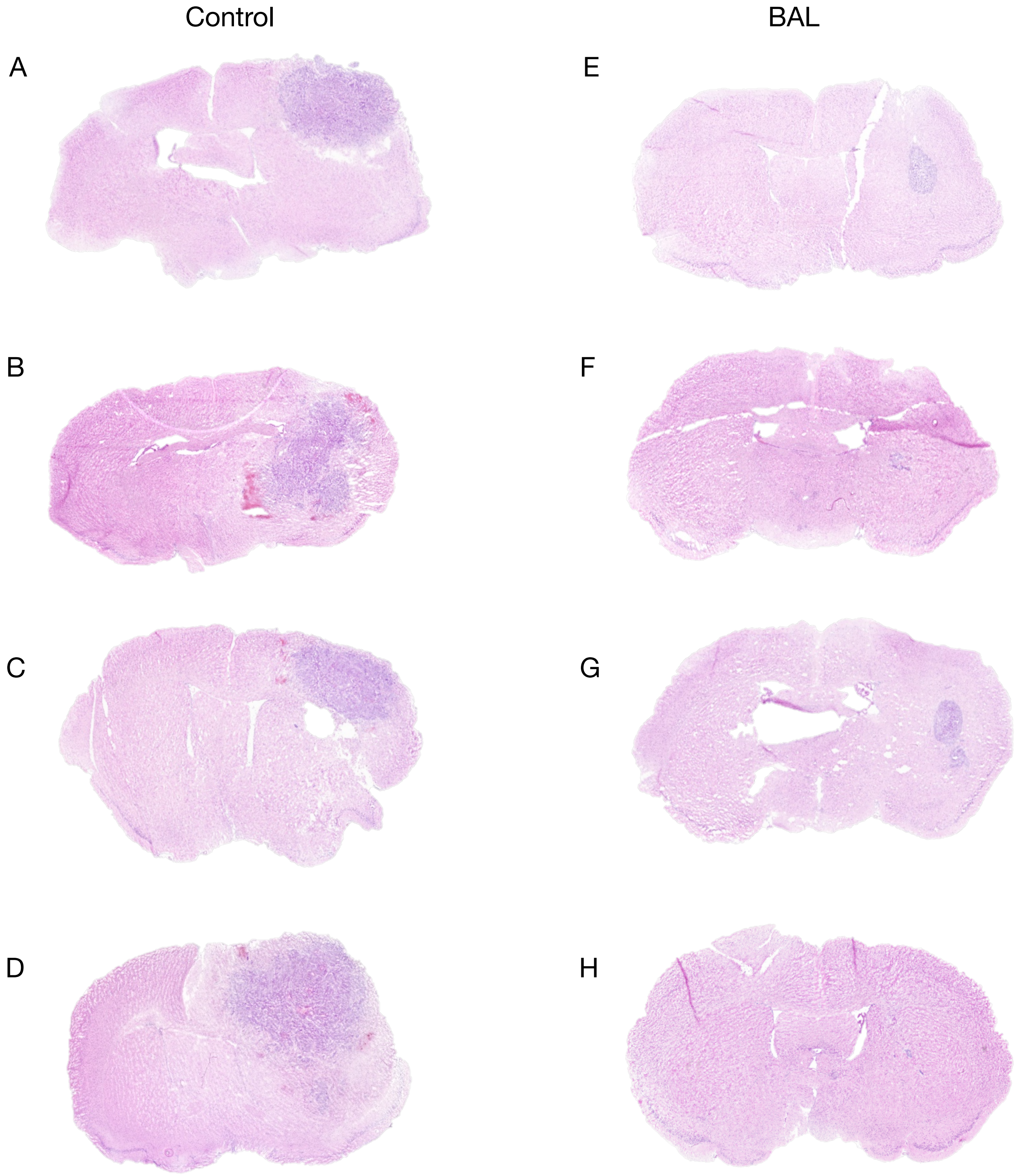

Supplementary Figure S3 – BAL101553 delays SB28 growth in vivo. (A - H) Representative pictures of coronal sections of brains from mice treated with vehicle control (A, B, C, D) or BAL101553 (BAL) (E, F, G, H) and stained with haemotoxylin and eosin at day 21 post intra-cranial implantation of SB28. Each section corresponds to a different mouse brain.

Supplementary Figure S4

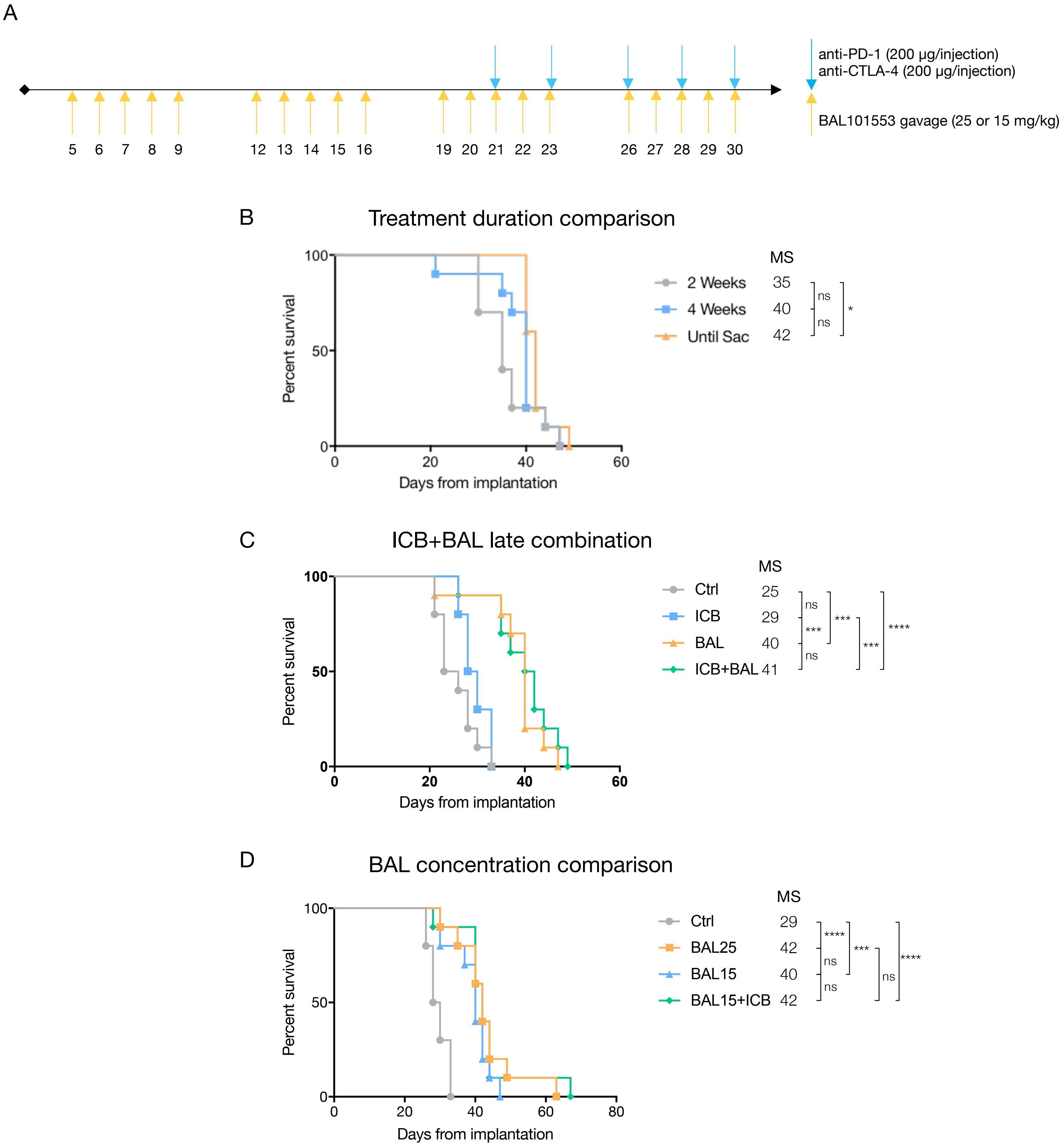

Supplementary Figure S4 – Low dose BAL101553 treatment is as efficient as higher dosage, but does not sensitize to immune checkpoint inhibitors. (A) Treatment schedule of mice intra-cranially implanted with SB28 at day 0. (B) Symptom-free survival curve of mice treated from day 5 post-implantation for 2 or 4 weeks or until terminal symptoms (5 out of 7 days per week). (C) Symptom-free survival curve of mice treated with BAL101553 (BAL) or a late immune checkpoint inhibitor (ICB) treatment, and the combination of both. (D) Symptom-free survival curve of mice treated 4 weeks with vehicle control (Ctrl), 25mg/kg of BAL1015553 (BAL25) or 15mg/kg of BAL101553 (BAL15) and in combination with ICB at late stage as shown in A (BAL15+ICB). Median survival (MS) is displayed in days. Statistics: Log-rank (Mantel-Cox): non-significant (ns) $p > 0.05$; *: $p<0.05$; ***: $p<0.001$; ****: $p<0.0001$.

Supplementary Figure S5

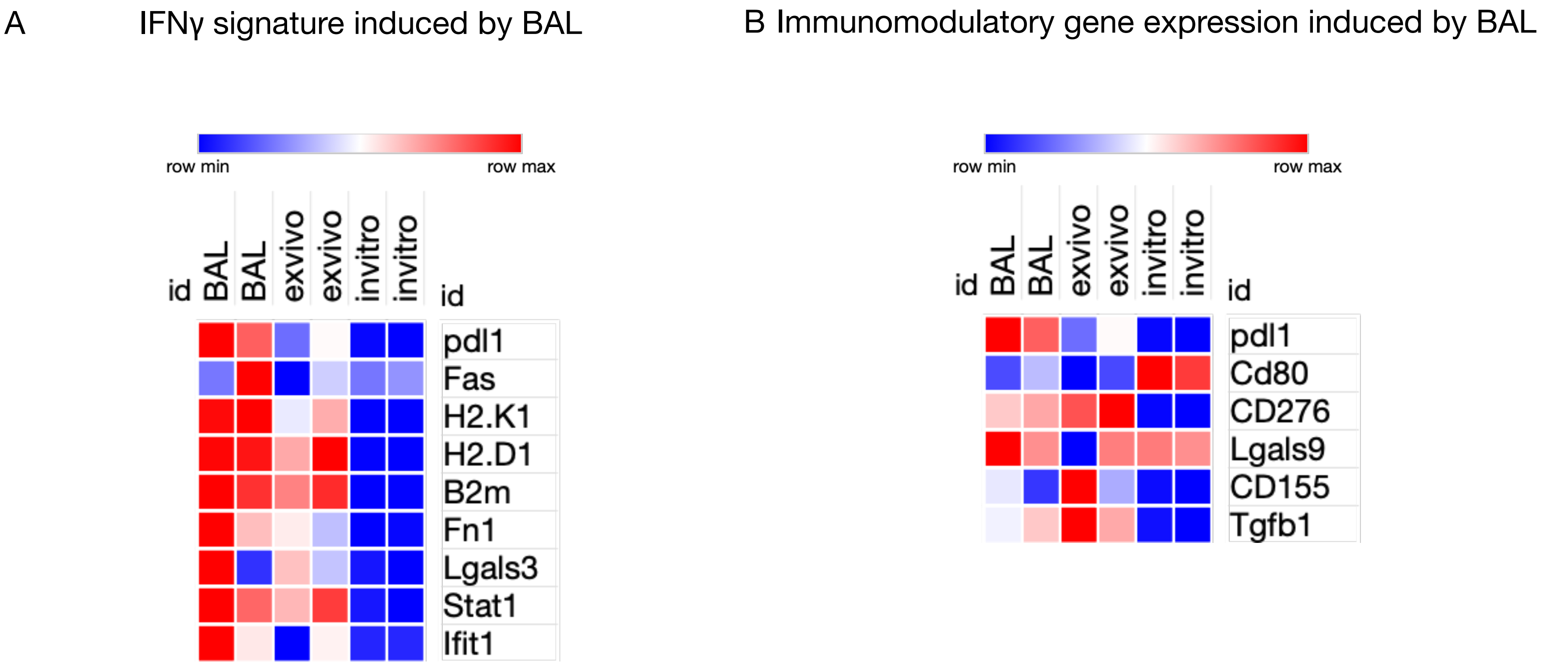

Supplementary Figure S5 – BAL101553 does not significantly impact on IFNγ gene signature or immunomodulatory gene expression by SB28 cells in vivo. (A) Gene expression analysis of genes comprising the interferon-γ (IFNγ) signature after growth of SB28 cells in vitro or from in vivo controls and BAL101553 (BAL) treated mice. (B) Gene expression analysis of immunoregulatory molecules based on RNA sequencing of SB28 cells growing in vitro or from in vivo controls and BAL101553 treated mice. All comparisons are statistically non-significant ($p>0.05$). Statistics: t-test corrected with false discovery rate (FDR).

Supplementary Figure S6

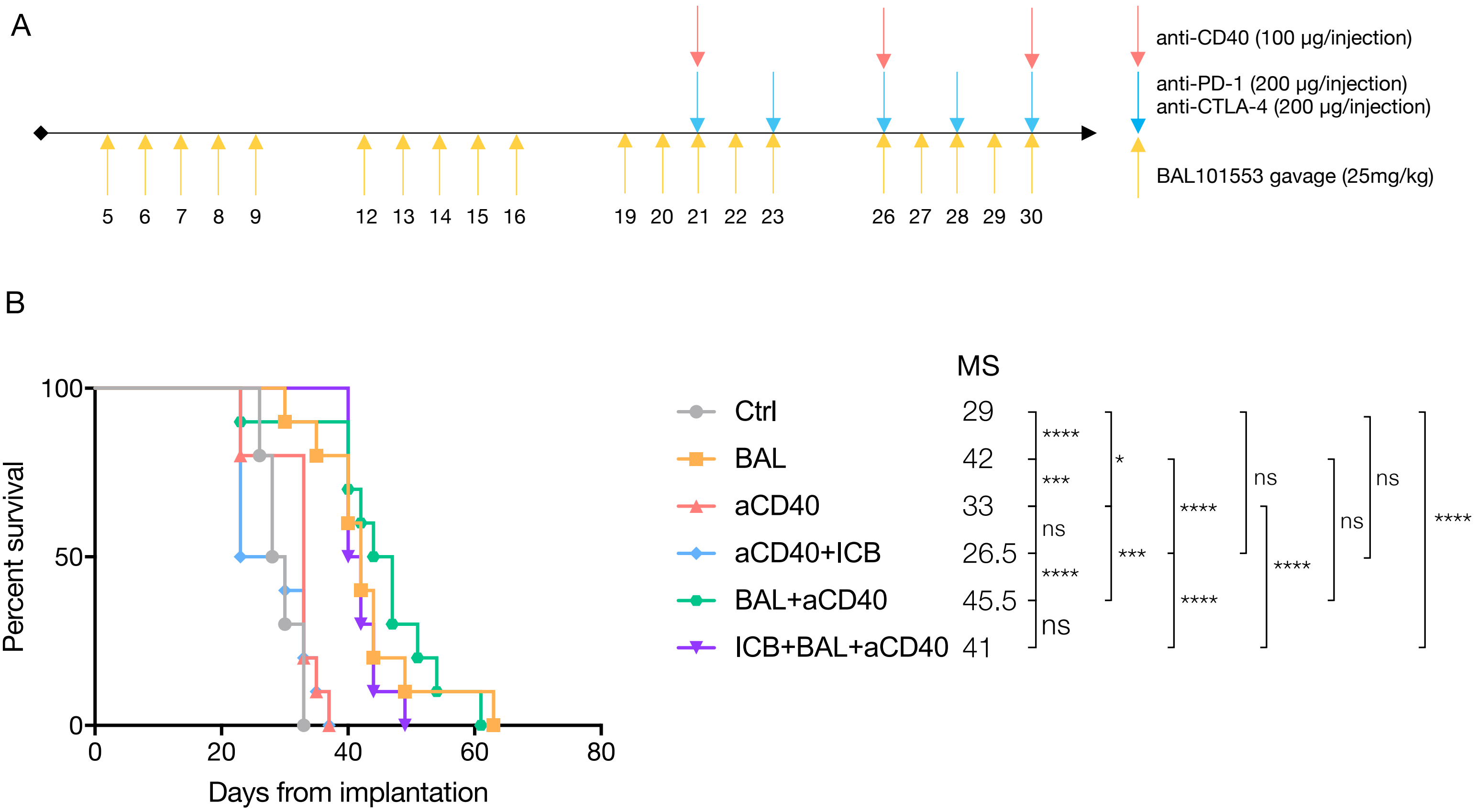

Supplementary Figure S6 – Addition of anti-CD40 to combined immune checkpoint blockade and BAL101553 therapy does not improve survival of SB28 implanted mice. (A) Treatment schedule of mice, intra-cranially implanted with SB28 at day 0. (B) Symptom-free survival curve of mice treated with vehicle control (Ctrl), agonistic anti-CD40 (aCD40), BAL101553 (BAL), a combination of BAL and aCD40 (BAL+aCD40), a combination of anti-PD-1 and anti-CTLA-4 (ICB) and aCD40 (aCD40+ICB) or a combination of all therapies (ICB+BAL+aCD40). Median survival (MS) is displayed in days. Statistics: Log-rank (Mantel-Cox): non-significant (ns) p> 0.05; *: p<0.05; ***: p<0.001; ****: p<0.0001. n=8-10 mice per group. n=10 mice per group.

Supplementary Figure S7

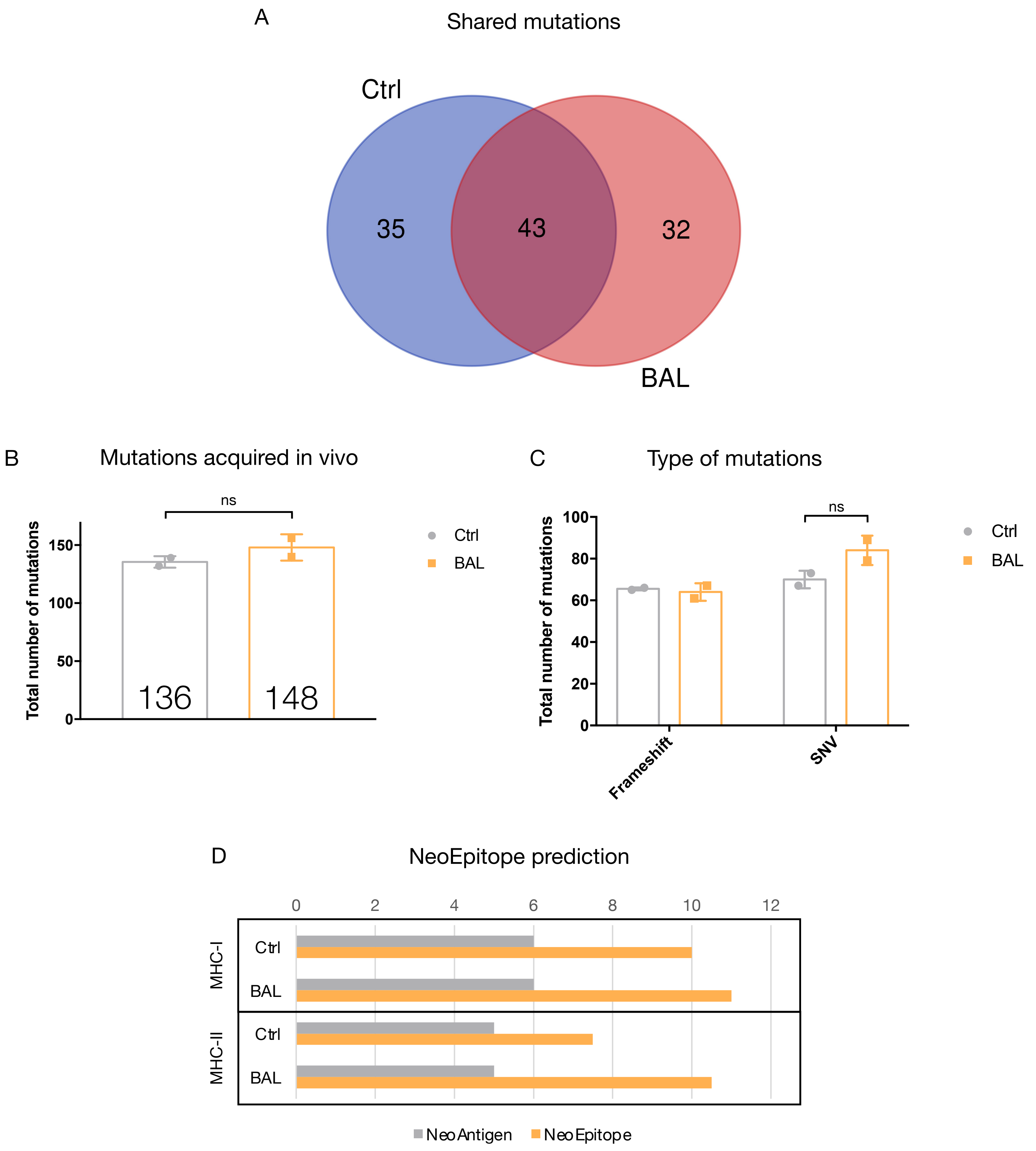

Supplementary Figure S7 – BAL101553 treatment does not significantly induce more mutations or generate more neoepitopes on SB28 tumor cells tested ex vivo . (A) Analysis of all identified mutations on SB28 cells ex vivo after vehicle control or BAL101553 (BAL) treatment, based on WES data shows 43 common mutations. (B, C) Whole exome sequencing data of SB28 cells from ex vivo control and BAL101553 treated mice, with total mutational load (B) and analysis of mutation type (C). (D) The same samples were analyzed by algorithms (see methods for details) to predict generation of neoepitopes for MHC-I and MHC-II. Statistics: Tukey's test corrected with false discovery rate (FDR); non-significant (ns): $p > 0.05$. 2 biological replicates are analyzed for each group.

Supplementary Figure S8

A

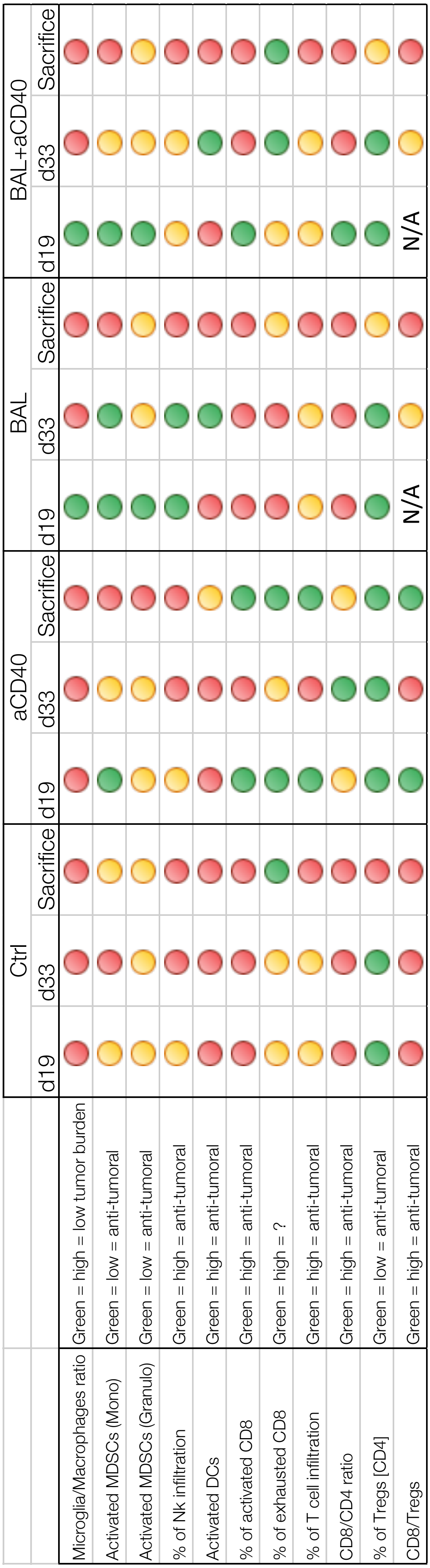

(legend on next page)

Supplementary Figure S8 (continued)

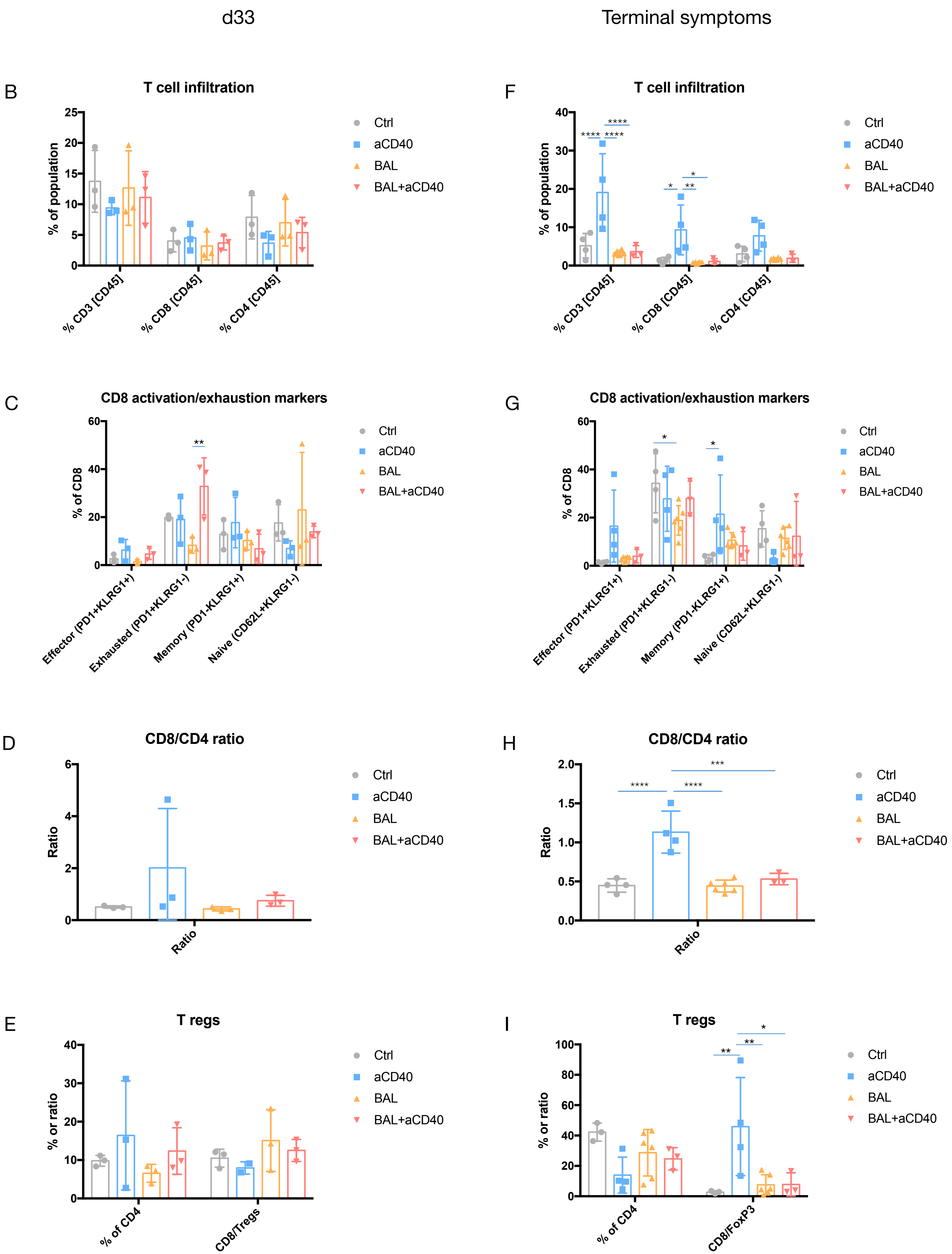

Supplementary Figure S8 (continued)

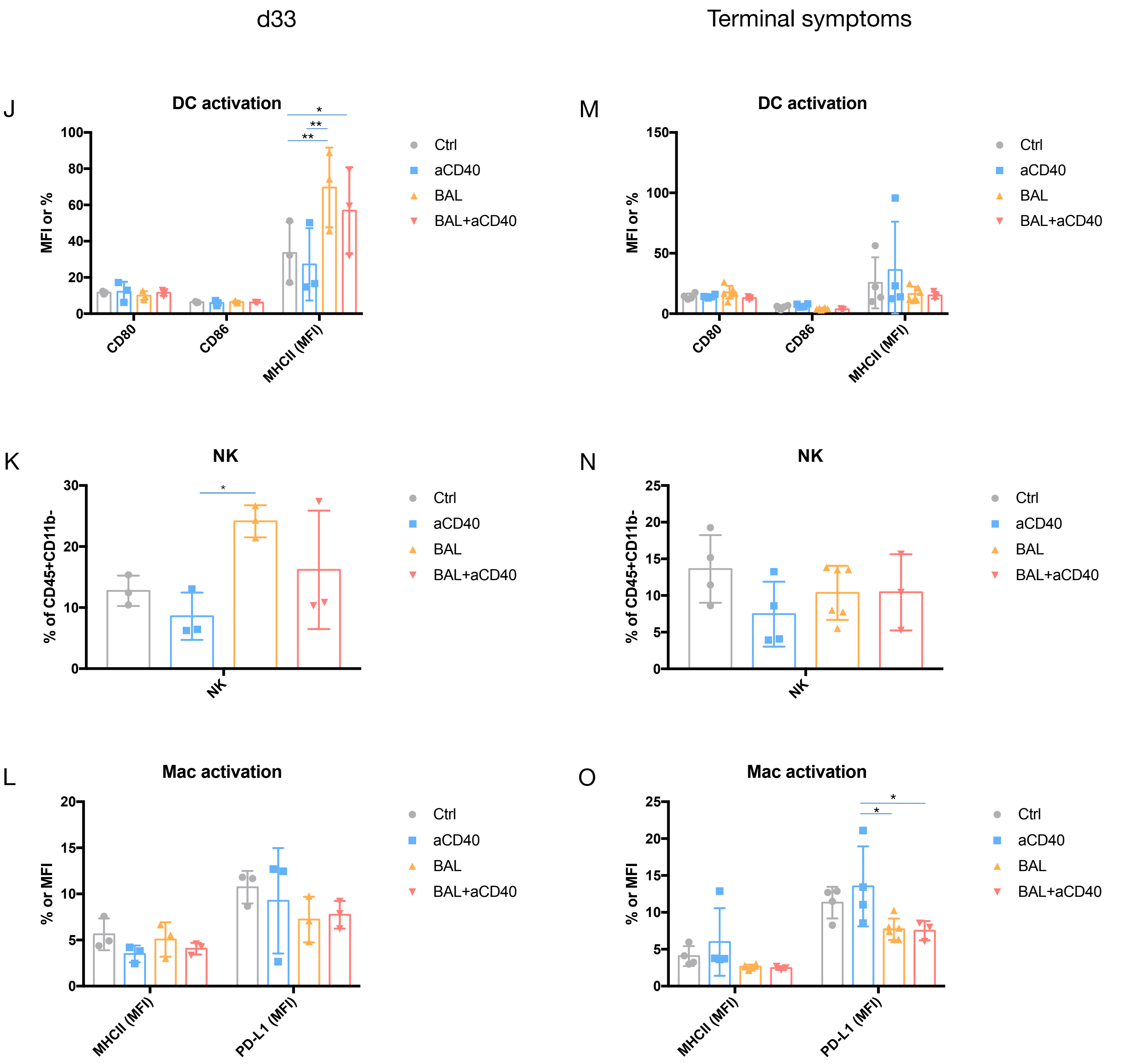

Supplementary Figure S8 –Brain infiltrating immune cells are impacted by the different treatment protocols. (A) Table summarizing many of the cell subtypes analyzed at d19 or 33 after tumor implantation, or at time of terminal symptoms and classified as green, orange or red for their prospected association with pro-tumoral, in between, or anti-tumoral impact respectively. (B-O) Brain infiltrating leucocytes from mice intracranially-implanted with SB28 and treated with vehicle control (Ctrl), anti-CD40 (aCD40), BAL101553 (BAL) or a combination of BAL and aCD40 were collected at d33 (B, C, D, E, F, G, H) or at time of terminal symptoms (I, J, K, L, M, N, O) and stained for subsequent flow-cytometry analysis, multiple immune cell subtypes are displayed. Statistics: Tukey's test, *: p<0.05; **: p<0.01; ***: p<0.001; ****: p>0.0001. Error bars indicate SD. N/A: non applicable. Cell populations analyzed are the following: T cells (CD3^{+}), CD8 T cell (CD3^{+} and CD8^{+}), CD4 T cells (CD3^{+} and CD4^{+}), leukocytes (CD45^{+}), Effector CD8 T cells (CD3^{+} and CD8^{+} and PD1^{+} and KLRG1^{+}), Exhausted CD8 T cells (CD3^{+} and CD8^{+} and PD1^{+} and KLRG1^{-}), Memory CD8 T cells (CD3^{+} and CD8^{+} and PD1^{-} and KLRG1^{+}), Naive CD8 T cells (CD3^{+} and CD8^{+} and CD62L^{+} and KLRG1^{-}), Regulatory T cells (CD3^{+} and CD4^{+} and FoxP3^{+}), DC (CD11b^{+} and CD11c^{+}), NK cells (NK1.1^{+}), Macrophages (CD11b^{+} and CD11c^{-}). Monocytic and Granulocytic MDSCs respectively (CD11b^{+} and LY6G^{+}) and (CD11b^{+} and LY6C^{+}). All cells are gated on CD45^{+} after exclusion of doublets and dead cells. % x [y] corresponds to the % of the x population gated on the y population.

Supplementary Figure S9

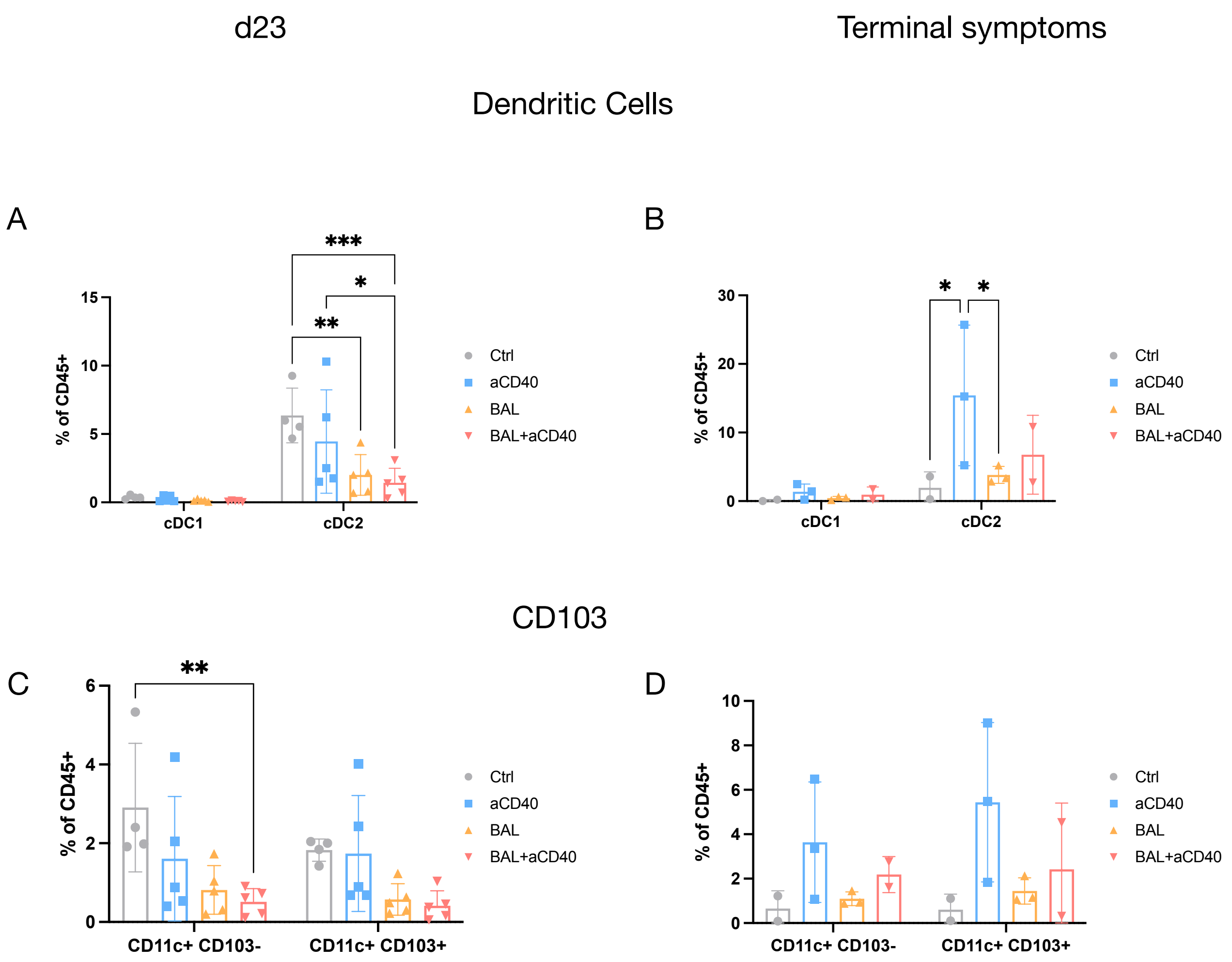

Supplementary Figure S9 – cDC2 are less infiltrating BAL treated mice implanted with SB28. (A, B) Proportion of brain infiltrating dendritic cells, and expression of CD103 by $CD11c^{+}$ cells positive (C, D), from mice intracranially-implanted with SB28 and treated with vehicle control (Ctrl), anti-CD40 (aCD40), BAL101553 (BAL) or a combination of BAL and aCD40. Brains were collected at d23 (A, C) or at time of terminal symptoms (B, D). Statistics: Tukey's test, *: $p<0.05$; ***: $p<0.001$; ****: $p<0.0001$. Error bars indicate SD. cDC1 are defined as ($CD11c^{+}CD8^{+}$), cDC2 as ($CD11c^{+}CD8^{-}$). All cells are gated on $CD45^{+}$ after exclusion of doublets and dead cells.

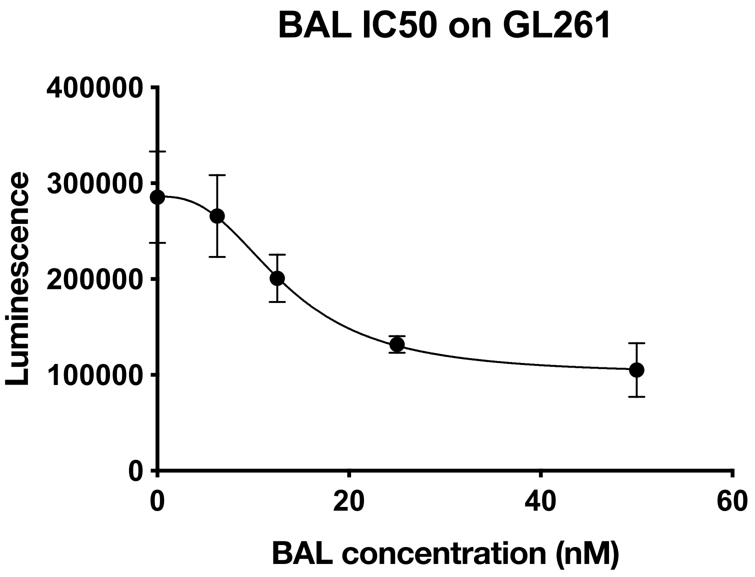

Supplementary Figure 10 – BAL27862 impacts on viable GL261 cell in vitro. Number of viable GL261 cells based on total ATP content by luminescent cell viability/proliferation assay; GL261 sensitivity to BAL27862 (BAL) corresponding to an IC50 of 13.42nM. Results include 3 biological replicates.
